# Global transcriptome analysis of the C57BL/6J mouse testis by SAGE: evidence for nonrandom gene order

**DOI:** 10.1186/1471-2164-6-29

**Published:** 2005-03-05

**Authors:** Petr Divina, Čestmír Vlček, Petr Strnad, Václav Pačes, Jiří Forejt

**Affiliations:** 1 Institute of Molecular Genetics, Academy of Sciences of the Czech Republic and Center for Integrated Genomics, Vídeňská 1083, CZ-142 20, Prague 4, Czech Republic

## Abstract

**Background:**

We generated the gene expression profile of the total testis from the adult C57BL/6J male mice using serial analysis of gene expression (SAGE). Two high-quality SAGE libraries containing a total of 76 854 tags were constructed. An extensive bioinformatic analysis and comparison of SAGE transcriptomes of the total testis, testicular somatic cells and other mouse tissues was performed and the theory of male-biased gene accumulation on the X chromosome was tested.

**Results:**

We sorted out 829 genes predominantly expressed from the germinal part and 944 genes from the somatic part of the testis. The genes preferentially and specifically expressed in total testis and testicular somatic cells were identified by comparing the testis SAGE transcriptomes to the available transcriptomes of seven non-testis tissues. We uncovered chromosomal clusters of adjacent genes with preferential expression in total testis and testicular somatic cells by a genome-wide search and found that the clusters encompassed a significantly higher number of genes than expected by chance. We observed a significant 3.2-fold enrichment of the proportion of X-linked genes specific for testicular somatic cells, while the proportions of X-linked genes specific for total testis and for other tissues were comparable. In contrast to the tissue-specific genes, an under-representation of X-linked genes in the total testis transcriptome but not in the transcriptomes of testicular somatic cells and other tissues was detected.

**Conclusion:**

Our results provide new evidence in favor of the theory of male-biased genes accumulation on the X chromosome in testicular somatic cells and indicate the opposite action of the meiotic X-inactivation in testicular germ cells.

## Background

From the selfish DNA perspective [1,2], gonads are fundamentally important organs of an organism. During the first meiotic division of gametogenesis, crossing-over enhances the re-assortment of information carried in parental DNA molecules and virtually immortal genetic information is then transferred to next generations of mortal individuals via the final products of gametogenesis, spermatozoa and eggs. Moreover, testes and ovaries are the only niches where the paternal and maternal DNA interacts with a different environment. The dissimilar gonadal environment enables sex-dependent epigenetic modifications of paternal and maternal DNA such as reactivation of the X chromosome in female germ cells [3,4], inactivation of a single X chromosome in pachytene spermatocytes [5-7] or differential establishment of imprinting marks on paternally or maternally imprinted genes [8,9]. Spermatogenesis also serves as an important checkpoint filtering out many *de novo *occurring gene mutations [10,11] and chromosomal rearrangements [12,13] by making their carriers sterile. A special form of meiotic checkpoint is represented by hybrid sterility, which facilitates creation of new species. Obeying the Haldane's rule, hybrid sterility preferentially affects gametogenesis in testis in species with heterogametic (XY) sex [13-15]. Molecular analyses of these phenomena are hindered by the fact that testis is a complex organ with many types of intimately intermingled somatic and germline cells. Moreover, the spermatogenic differentiation is almost impossible to achieve *ex vivo*, in a cell culture system. The main cell types can be fractionated, via gravity sedimentation, centrifugal elutriation or fluorescence activated cell sorting, but the time required can be fairly long to exclude possible artificial changes of mRNA levels.

In the present work we used Serial Analysis of Gene Expression (SAGE) [16] to characterize the transcriptome of mouse total testis. We created a catalogue of genes expressed in the adult mouse testis of the C57BL/6J (abbreviated here B6) inbred strain. The B6 inbred strain has been chosen because its genome has been recently sequenced [17] and since it has been selected as a recipient strain for creation of two sets of Chromosome Substitution Strains, C57BL/6J-Chr#^A/J ^[18] and C57BL/6J -Chr#^PWD/Ph ^[Gregorova S, Forejt J et al., in progress]. Except for the characterization of the total testis transcriptome, we compared our data with the publicly available SAGE library from adult testis somatic cells [19] and other SAGE libraries constructed from normal mouse tissues. Furthermore, we were interested in the organization of testicular genes in the mouse genome and we present here a detailed bioinformatic analysis of the distribution of testicular genes between the X chromosome and autosomes, and the positional clustering of genes with preferential expression in testis.

## Results

### Characterization of the SAGE libraries of B6 mouse testis

**Figure 1:**
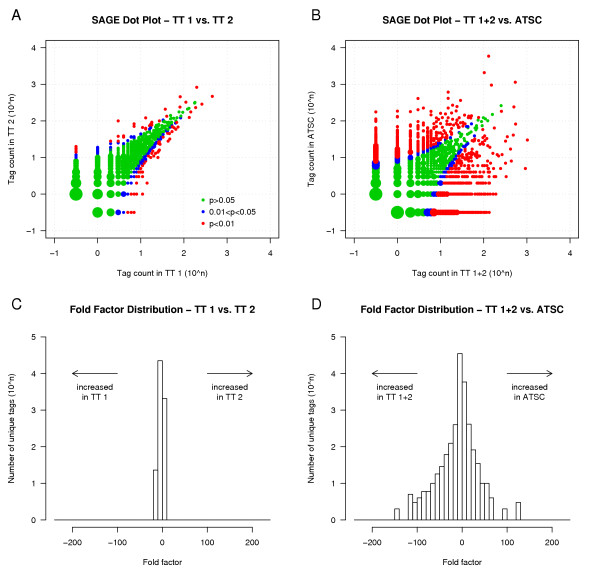
**Comparison of mouse testis SAGE libraries represented by dot plots and fold factor distribution graphs. **Comparison of tag counts between two total testis libraries (A), and between the combined total testis library and adult testis somatic cells library (B). Tags with significant p-chance are depicted in blue (0.01 < p < 0.05) and red (p < 0.01). Tags missing in one of the libraries are plotted at -0.5 coordinates. Point size is proportional to the number of represented tags. Distribution of the fold factor between two total testis libraries (C), and between the combined total testis library and adult testis somatic cells library (D). Fold factor is the ratio of normalized tag abundances in two SAGE libraries with ratios < 1 converted to reciprocal negatives. For tags missing in one library, normalized tag count of single copy tags was assumed. Abbreviations: TT 1 = total testis library 1; TT 2 = total testis library 2; TT 1+2 = combined total testis libraries; ATSC = adult testis somatic cells library.

We have constructed two high-quality SAGE libraries, TT 1 and TT 2, from the total mouse testis of adult B6 males (Table 1). The libraries contain 24 975 (TT 1) and 51 879 (TT 2) tags corresponding to 10 516 and 18 848 unique tags, respectively. The tags with abundance > 1 comprise 17 244 (69 %) and 38 457 (74 %) of the total tag mass but only 2 785 (26.5 %) and 5 426 (29 %) of the unique tags, respectively. The high average number of tags per clone (> 30) and low contamination with linker-derived tags (< 1 %) and duplicated ditags (~1%) indicate that the SAGE libraries are of high quality. Both total testis SAGE libraries provided similar gene expression profiles (R^2 ^= 0.84 for all unique tags, Pearson correlation), which suggests a good reproducibility of SAGE data. However, a certain variation was observed in the tag abundances when 24 529 unique tags found in both total testis SAGE libraries were compared by Monte Carlo simulations. Three hundred thirteen tags exhibited significant differences in their frequency between TT 1 and TT 2 libraries at p < 0.05 (89 tags at p < 0.01) representing non-hereditary variations in transcription profiles and variations introduced by the experimental process. The fold factor value (defined as the ratio of normalized tag counts in TT 2 to TT 1 libraries, with ratios < 1 converted to reciprocal negative values) for 93.5 % of the compared tags ranged between -2.2 and 2.2 (for 99% of the tags between -5 and 5). Dot plot comparison and fold factor distribution graphs (Fig. 1A,C) depict the similarity of both total testis libraries. Despite this variation, the SAGE method produced reproducible gene expression profiles and the libraries could be combined into the total testis SAGE library (referred to hereafter as TT 1+2) with the total of 76 854 tags and 24 529 unique tags. The raw data from the total testis SAGE libraries are deposited in the GEO repository [20] under accession numbers GSM34767 (TT 1) and GSM34768 (TT 2). The set of tags with abundance > 1 in TT 1+2 SAGE library with reliable tag identification is listed in Additional file 1. The testis SAGE libraries are also freely available for interactive exploration and analysis in the Mouse SAGE Site database [21].

**Table 1:** Parameters of constructed SAGE libraries from B6 mouse total testis

SAGE library	Total testis 1 (TT 1)	Total testis 2 (TT 2)
Sequenced clones	811	1 510
Total tags*	24 975	51 879
Unique transcripts	10 516	18 848
Single copy tags	7 731	13 422

Quality parameters		

Average tags per clone	30.8	34.4
Duplicated ditags	157 (1.2 %)	276 (1.0 %)
Linker derived tags	147 (0.6 %)	223 (0.4 %)

* excluding tags from duplicated ditags and linker-derived tags (i.e. two linker tags TCCCTATTAA, TCCCCGTACA and all possible 1-bp linker tag variations)

### Tag-to-gene identification in the B6 testis transcriptome

Tag-to-gene identification in the TT 1+2 SAGE library was evaluated using three different criteria applied to the SAGEmap database. The first was the most commonly used SAGEmap reliable mapping [22,23]. The second was a modified approach based on the SAGEmap full mapping file and implemented in the Mouse SAGE Site database [21]. In this approach, the tag-to-gene associations were considered reliable if supported by tags extracted from at least one mRNA sequence (from RefSeq, Mammalian Gene Collection or GenBank) or at least 3 ESTs with a poly(A) signal or at least 8 ESTs with no poly(A) signal [24]. The third approach (referred to here as RNA evidence mapping) was also based on the SAGEmap full mapping file. Tag-to-gene associations were considered reliable if supported by tags extracted from at least one mRNA sequence. The 7 481 tags with tag count > 1 in the TT 1+2 library were subjected to the SAGEmap reliable mapping that could identify 92.6 % tags to UniGene clusters (54.3 % to single and 38.3% to multiple genes; Table 2). When a more restricted reliable mapping from the Mouse SAGE Site was used, only 63% tags were identified to UniGene clusters (47.5% to single and 15.5 % to multiple genes) and about 29.6 % tags had unreliable identification to one or more UniGene clusters. Based only on the tags extracted from mRNA sequences, the RNA evidence mapping identified 51.3 % tags to UniGene clusters (45 % to single and 6.3 % to multiple genes) leaving 41.3% tags with unreliable identification. Using any of the tag identification methods, 7.4 % tags could not be identified to UniGene clusters and may be associated with novel genes. Further in this work, we used Mouse SAGE Site or RNA evidence mapping appropriately for a particular analysis (as indicated in Methods and Additional files).

**Table 2:** Identification of tags in the combined total testis SAGE library (TT 1+2) The tags matching the mitochondrial genome were omitted in this summary. Tags in group "Unreliable matches" (*) are considered not reliable according to Mouse SAGE Site and RNA evidence mappings, because they are not supported by the required number of mRNA and EST sequences. These tags are, however, included in reliable single/multiple match groups in the SAGEmap reliable mapping, which results in a highly increased number of reliable multiple matches and a slightly increased number of reliable single matches.

	NCBI SAGEmap reliable mapping		Mouse SAGE Site reliable mapping		RNA evidence mapping	
	
	tags	%	tags	%	tags	%
Reliable single match	4 061	54.3	3 553	47.5	3 367	45.0
Reliable multiple matches	2 865	38.3	1 157	15.5	472	6.3
Unreliable match(es)*	-	-	2 216	29.6	3 087	41.3
No match	555	7.4	555	7.4	555	7.4

Total tags (tag count > 1)	7 481	100.0	7 481	100.0	7 481	100.0

### Functional categories of genes expressed in total testis

**Figure 2:**
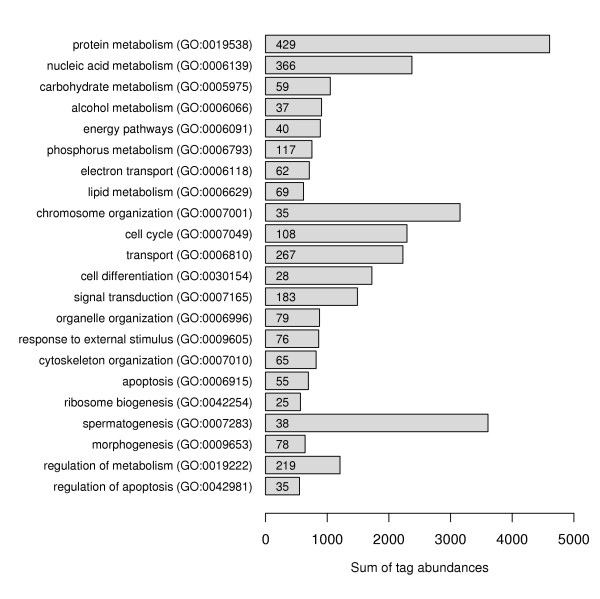
**Classification of genes expressed in total testis according to the biological process ontology of the GO database. **Bar graphs represent the sum of tag abundances corresponding to genes associated with a particular GO term. Only selected GO terms with the sum of tag abundances > 500 are displayed. The number of genes associated with each GO term is indicated inside the bars.

We associated genes and their corresponding tag counts to functional categories from the biological process ontology of GO database [25,26] (Fig. 2). In the total testis transcriptome, we observed more than 1000 genes involved in metabolism, particularly in the protein metabolism (protein modification, protein targeting) and nucleic acid metabolism (chromatin assembly and modification, DNA replication, DNA repair, RNA processing, RNA modification). As expected, the genes associated with spermatogenesis (e.g., protamine 1 and 2, transition proteins 1 and 2), chromosome organization, cell cycle and cell differentiation were highly expressed. Notably represented gene functions also included transport (e.g., diazepam binding inhibitor-like 5, proteasome 26S subunit, ribosomal protein L23), signal transduction (e.g., calmodulin 1 and 2, sperm autoantigenic protein 17, A kinase (PRKA) anchor protein 3, PDZ domain containing 1, WD repeat domain 12), cytoskeleton organization (e.g., t-complex testis expressed1, t-complex-associated testis expressed 3, tubulin alpha7/alpha 3, tubulin alpha 6, thymosin beta 10) and apoptosis (e.g., Bcl2-associated athanogene 1, Bcl2-like 14, programmed cell death 5, tumor protein translationally-controlled 1). From the mitochondrial genome, ATP synthase 6, cytochrome c oxidase I and III were the most highly expressed genes.

### Comparing the transcriptomes of total testis and adult testis somatic cells

The mouse testis is composed of two main cell types with principally different origin and functions, the germ cells that differentiate from spermatogonia to mature spermatozoa and the somatic cells that carry out all supportive functions to make the spermatogenesis and reproduction possible. Seminiferous tubules of the adult testis consist of approximately 88% germ cells and 12% somatic cells including myoid and Sertoli cells [27]. We compared our total testis SAGE library (TT 1+2) with a SAGE library constructed from the somatic cells of adult testis (GEO, accession GSM5435). This library was created from testes largely devoid of germ cells 60 days after busulphan treatment [19]. The SAGE library sizes are similar for TT 1+2 and the adult testis somatic cells (abbreviated here ATSC) comprising 76 854 and 81 478 tags, respectively. The number of unique tags (24 529 and 22 809) as well as the proportions of tags with abundance > 1 to the total tag mass (77.8% and 81.1 %) and to the number of unique tags (30.5 % and 32.6 %) are also comparable (Table 3). As anticipated, comparison of TT 1+2 and ATSC SAGE libraries using Monte Carlo simulations revealed extensive differences in gene expression between total testis and somatic cells of adult testis. Out of the 42 239 unique tags in TT 1+2 and ATSC libraries, the simulations detected significantly different tag abundances in 3 258 tags at p < 0.05. Concerning the fold factor, 83 % of the compared tags stretch in the range between -2.2 and 2.2 (92.5% tags between -5 and 5). At the extreme ends, 563 tags reach > 10-fold increase in tag counts in the ATSC library (fold factor > 10) and 672 tags reach > 10-fold increase in the TT 1+2 library (fold factor <-10) (see Additional file 2). Dot plot comparison and fold factor distribution graphs for TT 1+2 and ATSC transcriptomes illustrate their dramatic dissimilarities (Fig. 1B,D).

**Table 3:** Parameters of the SAGE libraries constructed from total testis and somatic cells of adult testis

SAGE library	Total testis	Adult testis somatic cells
	
	TT 1+2	ATSC
Total tags	76 854	81 478
Unique tags	24 529	22 809
Unique tags with count > 1	7 481	7 435

Proportions of unique tags with count > 1		

% of total tags	77.8	81.1
% of unique tags	30.5	32.6

### Genes with predominant expression in the germinal or somatic component of testis

To sort out subsets of genes with predominant expression in either germinal or somatic cells of testis we applied tentative criteria to account for the presence of somatic cells in TT 1+2 and for residues of germ cells in ATSC. Predominant expression of a gene was considered if the corresponding tag was significantly more frequent in one of the libraries (p < 0.05, Monte Carlo simulations) and exhibited at least fivefold enrichment of tag counts (fold factor <-5 or > 5). According to this criterion a set of 829 genes is expressed predominantly in germ cells and 944 genes are expressed mainly from the somatic part of the testis (see Additional file 3). Moreover, we identified 12 tags corresponding to 8 genes encoded in the mitochondrial genome (1 gene with increased tag counts in TT 1+2 and 6 genes with increased tag count in ATSC). A gene coding for cytochrome c oxidase III (*mt-Co3*) displayed two tags separated by 87 bp in *mt-Co3 *gene mRNA. One isoform was predominantly present in the ATSC library and the other was observed exclusively in the TT 1+2 library. Substantial over-expression of mitochondrial cytochrome c oxidase complexes I, II, III and NADH dehydrogenase 3 and 4 was noted in testicular somatic cells (see Additional file 3).

### Exploring the dissimilarity of testis transcriptomes and transcriptomes of other mouse tissues

**Figure 3:**
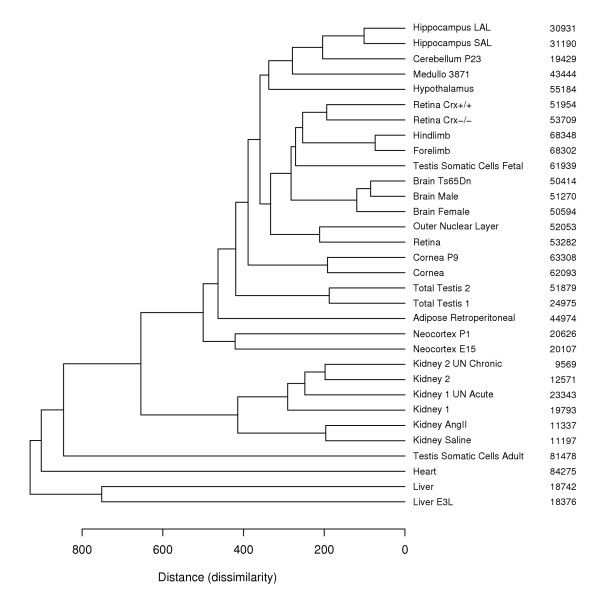
**Dissimilarities of mouse SAGE libraries illustrated by a dendrogram. **Thirty-two SAGE libraries constructed from bulk tissues containing 190 871 unique tags (including single copy tags) were selected (see Additional file 4). Pairwise library distances based on differences between normalized tag counts were computed according to [28]. The average agglomeration method was used in hierarchical clustering due to the highest cophenetic correlation (0.936) between observed and predicted distances resulting from the dendrogram. The number of tags in each SAGE library is indicated.

We examined the similarity of B6 testis transcriptomes to other available mouse SAGE transcriptomes created from normal and diseased bulk tissues by hierarchical clustering. Thirty-two SAGE libraries containing 190 871 unique tags (including single copy tags) were used as input in this analysis (see Additional file 4). We computed pair-wise library distances based on differences between normalized tag counts [28] and used the average agglomerative method for hierarchical clustering due to the highest cophenetic correlation (0.936). In the dendrogram of dissimilarities the two total testis SAGE libraries, TT 1 and TT 2, cluster together in contrast to the library from somatic cells of the adult testis (Fig. 3). The ATSC library is located separately and close to the libraries created from heart, liver and kidney in accord with the somatic origin of all these tissues. Interestingly, another SAGE library created from somatic cells of the fetal testis did not cluster with the ATSC library, but was placed close to the libraries from developing limbs, juvenile retina and whole brains. Another cluster consists of the six libraries generated from the whole adult kidneys. Several specialized brain tissues form a cluster with a brain tumor tissue (cerebellum, hippocampus, hypothalamus, medulloblastoma). An additional small cluster groups three libraries created from whole brain samples (normal male, trisomic Ts65Dn male and normal female).

### Nonrandom representation of testis-expressed genes on the X chromosome

Previous works have shown a significant enrichment of prostate- and spermatogonia-specific genes on the X chromosome when compared to autosomes [29,30]. We asked what proportion of testis-expressed genes maps to the X chromosome and compared it with the proportion of X-linked genes expressed in somatic (non-testis) tissues. Furthermore, we examined whether the proportion of testis-specific genes on the X chromosome differs from the proportion of X-linked tissue-specific genes in somatic tissues.

Out of the 14 222 genes expressed in SAGE libraries from total testis, adult testis somatic cells and 7 somatic tissues (brain, eye, heart, liver, kidney, limbs and adipose tissue) (see Additional file 4) we considered only genes identified by corresponding tag count > 1. The proportion of genes expressed from the X chromosome in a pool of 7 somatic tissues was 3.1 % (374 of 11 903 genes). Although the proportions of X-linked genes in somatic tissues were uneven, there were no significant differences among the tissues (3.2 % in brain, 2.7 % in limbs and eye, 2.6 % in liver, 2.5 % in kidney and adipose tissue, 2.4 % in heart; p > 0.05, Chi-square test for brain vs. heart). In testicular somatic cells, we observed 3.2% X-linked genes (133 of 4 216 genes), while in total testis only 1.4 % genes (48 of 3 338 genes) were expressed from the X chromosome (p < 10^-6^, Chi-square test). We can conclude that the number of expressed X-linked genes is underrepresented in the transcriptome of total testis.

The same set of 14 222 genes was examined for the distribution of tissue-specific genes on autosomes and the X chromosome. We compared the genes specific for either total testis (Table 4, a) or adult testis somatic cells (Table 4, b) in conjunction with somatic (non-testis) tissue-specific genes. A gene was considered to be tissue-specific if it was expressed only in one tissue type (total testis or adult testis somatic cells, brain, eye, heart, liver, kidney, limbs and adipose tissue). Moreover, the corresponding tag count > 1 was required to guarantee that the gene is truly expressed. The tissue-specific genes were assigned to chromosomes according to the LocusLink database and the significance of their chromosomal distribution was evaluated by permutations (see Methods) and confirmed by Fisher's exact test (Table 4). Out of the 395 genes specific for total testis 3.5% mapped to the X chromosome (see Additional file 5). Essentially the same proportion of X-linked genes was found for genes specific for 7 somatic (non-testis) tissues. In testicular somatic cells, we detected only 81 tissue-specific genes, but 13.6% were X-linked (see Additional file 5). This is a 3.2-fold increase in the proportion of testis somatic cell-specific genes on the X chromosome and represents their significant enrichment (p = 0.0024, two tailed, 100 000 permutations) in comparison to the genes specific for other tissues. All the X-linked testis-specific genes were subjected to BLAST against the whole X chromosome, which revealed no duplicated genes. The results from the permutation analysis indicate a significantly increased amount of testis-specific genes on the X chromosome in somatic cells of the testis when compared to autosomal testis-specific genes. The genes specific for 7 somatic tissues did not show a significant preference for the X chromosome. The list of X-linked genes expressed in total testis and testicular somatic cells with indicated testis-specific genes is available in Additional file 6.

**Table 4:** Distribution of testis-specific genes on autosomes and the X chromosome The total of 14 222 LocusLink genes were identified in total testis, adult testis somatic cells and non-testis tissue SAGE libraries (see Additional file 4) using RNA evidence mapping (tags matching multiple LocusLink genes were discarded). The genes identified by total tag count = 1 were then excluded from analysis. The genes expressed only in one tissue type (total testis, adult testis somatic cells, brain, eye, heart, liver, kidney, limbs and adipose tissue) were considered to be tissue-specific genes. Chromosomal distribution of genes specific for total testis (a) and testis somatic cells (b) in comparison to the non-testis tissue-specific genes was evaluated. The significance was tested by permutations (100 000 random shufflings of the chromosomes while keeping the sum of genes on autosomes and the X chromosome fixed) and confirmed by Fisher's exact test. Abbreviations: total t. = total testis; t. somatic = testicular somatic cells; other = non-testis tissues; ChrA = autosomes; ChrX = X chromosome.

a) Total testis: 395 genes specific for the combined total testis SAGE library (TT 1+2) Other tissues: 877 genes specific for one tissue type in the pool of other SAGE libraries							
Chrom	Observed gene counts		Gene counts in randomized genome		% observed gene counts		Ratio of observed proportions
	
	total t.	other	total t.	other	total t.	other	total t./other

ChrA	381	836	378	839	96.5	95.3	1.0
ChrX	14	41	17	38	3.5	4.7	0.7

							

Permutations yielding < = observed gene counts in total t. on ChrX							22 395
Permutations, p-value (two tailed)							0.4479

Fisher's exact, p-value (two tailed)							0.4563
Confidence interval (0.95)							0.70 – 2.68

b) Testis somatic cells: 81 genes specific for the adult testis somatic cells SAGE library (ATSC) Other tissues: 924 genes specific for one tissue type in the pool of other SAGE libraries							

Chrom	Observed gene counts		Gene counts in randomized genome		% observed gene counts		Ratio of observed proportions
	
	t.somatic	other	t.somatic	other	t.somatic	other	t. somatic/other

ChrA	70	885	77	878	86.4	95.8	0.9
ChrX	11	39	4	46	13.6	4.2	3.2

							

Permutations yielding > = observed gene counts in t. somatic on ChrX							121
Permutations, p-value (two tailed)							0.0024

Fisher's exact, p-value (two tailed)							0.0013
Confidence interval (0.95)							0.13 – 0.64

### Chromosomal clustering of genes with preferential expression in testis

Based on the data from testis and other publicly available SAGE libraries (see Additional file 4) we identified genes with preferential expression in testis by Preferential Expression Measure (PEM) [31]. PEM score controls for the genes that are highly expressed in many tissues (housekeeping genes) and reports positive values for over-expressed genes and negative values for under-expressed genes in a given tissue. Large positive PEM scores for a gene in a particular tissue indicate that the gene is unusually highly expressed in that tissue, relative to its expression in other tissues [31]. We considered a gene to be preferentially expressed if the PEM score reached at least 50 % of the maximum PEM value encountered in that tissue. Using this criterion, we scored expression of genes in total testis or testicular somatic cells in conjunction with their expression in 7 other tissues (brain, eye, heart, liver, kidney, limbs and adipose tissue).

**Figure 4:**
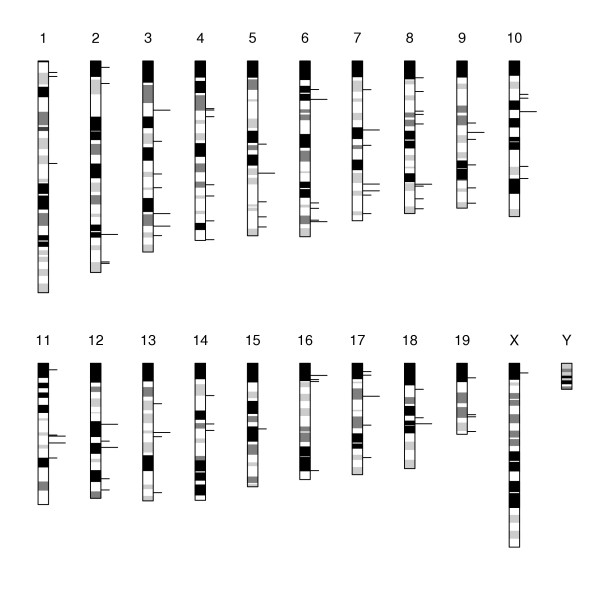
**Chromosomal positions of clusters containing preferentially expressed genes in testis. **The positions of 103 gene clusters according to the physical map are displayed on an ideogram with corresponding cytogenetic bands on chromosomes. The clusters revealed in total testis and testicular somatic cells are not distinguished. Tight clusters (long dashes) form a subset of loose clusters (short dashes).

Further we analyzed the genome organization of genes preferentially expressed in testis. We evaluated the expression of 14 222 genes among the studied tissues and for 12 331 genes we were able to assign a genomic position according to the NCBI mouse genome assembly (build 32, mapping 19 684 known LocusLink genes). The genomic position was resolved for 5 252 and 5 843 genes expressed in total testis and testicular somatic cells, respectively, including 1 438 (27.4%) and 1 197 (20.5%) preferentially expressed genes, respectively (see Additional file 7). To evaluate the gene order of preferentially expressed genes in testis and to eliminate the effect of tandem duplications we purged the whole mouse genome of tandemly duplicated genes (see Methods). The tandem duplicate-free genome resulted in total of 16 858 LocusLink genes and preserved 1 300 and 1 050 genes preferentially expressed in total testis and testicular somatic cells, respectively. Using a search with a sliding window (see Methods) we localized chromosomal clusters containing at least three adjacent preferentially expressed genes (tight clusters). Similarly, we searched for clusters with at least three preferentially expressed genes among the six adjacent genes (loose clusters) to include genes that could be preferentially expressed but did not pass the above criterion for preferential expression or their expression was not detected by SAGE. By definition, the tight clusters form a subset of the loose clusters. The chromosomal distribution of clusters with preferentially expressed genes in testis is illustrated in Figure 4. We observed 44 and 36 genes preferentially expressed in total testis and testicular somatic cells located in 13 and 11 tight clusters, respectively. Two hundred and thirty and 120 genes preferentially expressed in total testis and testicular somatic cells resided in 66 and 37 loose clusters, respectively (Table 5; Additional file 8). Two of the tight clusters and eight of the loose clusters shared preferentially expressed genes between total testis and testicular somatic cells. Statistical analysis revealed that the observed number of preferentially expressed genes located in tight clusters was 2.0-fold and 3.1-fold higher for total testis and testicular somatic cells, respectively, than the average number of preferentially expressed genes located in clusters in randomized genomes (p = 0.0074 and p = 0.0005, one tailed, 100 000 permutations). Although only slighly higher (1.4- and 1.3-fold) than the average in randomized genomes, the observed number of preferentially expressed genes in testis located in loose clusters was still significant in case of total testis and nearly significant in case of testicular somatic cells (Table 5). Not surprisingly, the most highly expressed genes detected in total testis and involved in spermatogenesis (protamine 1, 2, 3 and transition protein 2) formed one of the tight clusters on chromosome 16. The results indicate a nonrandom distribution of the genes preferentially expressed in total testis and testicular somatic cells into chromosomal clusters, which did not arise from tandem duplications.

**Table 5:** Number of preferentially expressed genes in testis located in clusters within tandem duplicate-free mouse genome Out of the 19 684 known genes (LocusLink) mapped on mouse genome assembly (NCBI, build32), 16 858 genes remained in tandem duplicate-free genome, including 1 300 and 1 050 preferentially expressed genes in total testis and testicular somatic cells, respectively. Chromosome search found clusters containing at least three adjacent preferentially expressed genes (tight clusters) or at least three preferentially expressed genes among the six adjacent genes (loose clusters). The tight clusters therefore form a subset of the loose clusters. Observed gene counts were evaluated using permutations (100000 random shufflings of the expression status of genes while keeping the gene positions constant) and the average number of genes located in clusters in the randomized genomes was computed.

	Total testis (TT 1+2)		Adult testis somatic cells (ATSC)	
	
	1 300		1 050	
	
	in tight clusters	in loose clusters	in tight clusters	in loose clusters
Observed gene counts	44	230	36	120
Proportion of preferentially expressed genes	3.4 %	17.7 %	3.4 %	11.4 %
Gene counts in randomized genomes (mean ± std. dev.)	21.9 ± 8.1	168.4 ± 20.1	11.7 ± 5.9	94.2 ± 15.6
Ratio observed/mean in randomized genomes	2.0	1.4	3.1	1.3
Permutations yielding > = observed gene counts	741	180	52	5 722
p-value (one tailed)	0.0074	0.0018	0.0005	0.0572

### Comparing the B6 and BDF1 total testis transcriptomes

In a recent study focused on senescence changes in testis, a modified SAGE method was used to generate digital gene expression profiles of total testis from 3- and 29-month-old mice of the BDF1 strain and 14-month-old mice of the SAMP1 strain that exhibits an accelerated senescence [32]. Because of the different anchoring enzyme (*Rsa*I) used in construction of the libraries and the limited availability of data from the BDF1 testis transcriptome, we could perform only a rough manual comparison of our B6 testis transcriptome (76 854 tags) and the combined BDF1 testis transcriptome from 3- and 29-month-old BDF1 mice (41 221 tags). We focused on the most highly expressed testicular genes in GNF Mouse Atlas v2 [33,34] that were detected by Affymetrix GeneChips. A set of 35 highly expressed genes in testis (average difference > 9 000) was organized with SAGE tag counts from B6 and BDF1 testis (see Additional file 9). In the B6 total testis, we detected 33 out of 35 genes (the *Serf1 *gene could not be distinguished because its low complexity tag matches multiple genes and the *Cox7a2 *gene is not detected because its transcript lacks *Nla*III restriction site). In contrast, only 9 genes were detected in the BDF1 testis library, 13 genes were missing due to the absence of *Rsa*I restriction site in the transcript and for 13 other genes the expression data from BDF1 testis were not publicly available. Furthermore, out of the 35 highly expressed genes in testis, 21 genes were among the top 100 most expressed genes in the B6 total testis library, but only 9 genes were among the top 100 most expressed genes in the BDF1 total testis library. It appears that our SAGE data from the B6 testis transcriptome shows better correspondence to the microarray data than the data from the transcriptome of BDF1 testis.

## Discussion

Serial analysis of gene expression is a high-throughput method for building a catalogue of expressed genes and their expression levels of "normal" as well as diseased or genetically variant tissues and organs [16]. The digital character of SAGE data enables addition and direct comparison of different SAGE libraries, provided they were built with the same anchoring enzyme and originated from individuals of the same species. The utilization of such global transcriptome databases is multifold, including positional cloning of mutations or quantitative trait loci [35,36], functional genome annotation [37,38] or analysis of a nonrandom gene order [39]. Admittedly, the SAGE, as used in this work, has several limitations, including a significant proportion of repetitive and low complexity tags. The SAGE is obviously more labor-intensive than transcriptome analysis based on microarrays. At present, some of these inconveniencies can be solved by applying LongSAGE or massively parallel signature sequencing technologies [38,40].

In this study we constructed a SAGE library of the total testis of the C57BL/6J (B6) mouse inbred strain, compared it with other public available mouse SAGE libraries and analyzed localization of testis-expressed genes within the mouse genome. The B6 strain was favored for the availability of its high-quality draft genomic sequence [17] and because series of congenics and recently also consomic strains have used the B6 strain as a background strain [18] [Gregorova S, Forejt J, personal communication]. The combined total testis SAGE library, TT 1+2, consisted of 76 854 total tags representing 24 529 unique tags. The tag-to-gene reliable identification method used in Mouse SAGE Site [24] was applied to tags with frequency ≥ 2. Out of these tags, 47.5% (3 553) revealed a reliable match to single and 15.5% (1 157) to multiple UniGene clusters. Considering the size of the total testis SAGE library, medium to highly expressed genes are present in the expression profile. The library size is comparable to the recently published SAGE library of somatic cells of the mouse testis [19] and almost twice the size of a library constructed from the total testis of BDF1 hybrid mice using a modified SAGE method [32].

Contrary to microarrays, SAGE data are platform independent, which permits the use of unrelated datasets coming from various sources to compare gene expression patterns. We analyzed the mouse testis transcriptome by comparing our total testis SAGE library to the adult testis somatic cells library [19] and to additional publicly available SAGE libraries from 7 different tissues. We recognized three different modes of differential expression. (1) Predominant expression of genes in the germinal or somatic part of the testis, which did not consider expression in other tissues. (2) Preferential expression in testis that was defined by comparing the expression of testis to 7 somatic tissues for which SAGE data were available. (3) Testis-specific expression that was defined by null expression (at the resolution of a particular SAGE library) in SAGE libraries of seven tissues or organs other than testis. Complete lists of genes predominantly expressed in germinal or testis-somatic cells, as well as the catalogues of genes preferentially expressed in testis and testis-specific genes are available online in Additional file 3 , 5 and Additional file 7.

Conflicting results have been reported on the representation of male-biased genes on the X chromosome in various species. Spermatogonia-specific genes were found to be an order of magnitude more abundant on the mouse X chromosome [30]. In human, the prostate-specific genes were twice more frequent on the X chromosome, but the female mammary gland- and ovary-specific X-linked genes were not enriched in respective SAGE libraries [29]. On the contrary, under-representation or absence of male-biased genes on the X chromosome was reported in *Caenorhabditis elegans *[41] and in *Drosophila *[42,43]. In the mouse, an under-representation of testis-expressed and testis-enriched genes on the X chromosome was also revealed by the analysis of microarray and EST data [5-7]. Our present data favor under-representation of X-linked genes in the total testis transcriptome but not in testis-somatic cells. Because the germ cells in different stages of differentiation constitute about 90% of the total cell mass of testis, the data indicate that the deficit of X-linked testis-expressed genes may reflect the lack of transcription from the X chromosome in meiotic cells. These results are in agreement with the idea of X-chromosome silencing during the first meiotic division, the phenomenon based mostly on circumstantial evidence in flies and mice [7,44-46]. Thus, transcription at the haploid stage of spermatogenesis is expected for most of the X-linked genes expressed in total testis. The meiotic X chromosome inactivation seems to be restricted to primary spermatocytes, but Sertoli cells, which form the somatic part of seminiferous tubules, may have the X chromosome in the active state. Indeed, in the transcriptome of adult testis somatic cells the proportion of expressed X-linked genes (3.2 %) was more than twice higher than in total testis (1.4 %) and did not differ from the proportion of X-linked genes expressed in non-testis (somatic) tissues.

Testis-specific genes belong to a wider category of sex-biased genes, which according to the hypothesis of sexually antagonistic genes are more likely to spread on the X chromosome than on autosomes [47]. This is because on the X chromosome they will express their favorable effect in the hemizygous state (XY) while their deleterious effect will be masked by their recessivity in the other sex (XX). Consequently, accumulation of male-specific genes on the X chromosome will be possible by the effect of modifiers that narrow the expression of sex-biased genes only to the male sex [47]. Thus, the evolution of sexually antagonistic genes and X inactivation may act as opposing forces on the germline lineage of testis while accumulation of male-specific genes could be expected in somatic cells of testis. In accord with these assumptions the proportion of X-linked genes specific for total testis did not significantly differ from the proportion of genes specific for other tissues, while we observed a significant 3.2-fold enrichment of the proportion of X-linked genes specific for testicular somatic cells.

The eukaryotic gene order is nonrandom obviously not only due to shifting of sex-biased genes to and from the X chromosome, but also owing to a nonrandom clustering of genes within chromosomes. This somewhat unexpected conclusion (taking into account the relative autonomy of transgene regulation) is gaining gradual support from global transcriptome analyses of various eukaryotic species (see Hurst et al. for review) [39]. The observed examples of clustering are apparently a mixture of several unrelated phenomena, including large domains of similarly expressed genes in *Drosophila *and humans [48,49], clustering of housekeeping genes [50], clustering of highly expressed genes [51] or genes with similar expression breadth in regions of similar GC content [52]. In *Drosophila melanogaster *one third of testes-specific genes occur in clusters [43], a phenomenon not reported in any other species. Using PEM [31] to define preferentially expressed genes we were able to demonstrate that in the mouse, the genes preferentially expressed in germ cells as well as in somatic cells of testis occur in tight clusters with a frequency 2.0-fold and 3.1-fold higher than the expected average frequency in randomized genomes. Moreover, our results indicate that this phenomenon is not merely a consequence of tandem duplications. Further analysis of clustering of testis-expressed genes may reveal new insights into the functional organization of the mammalian genome.

## Conclusion

We identified chromosomal clusters of adjacent genes with preferential expression in testis that contain a significantly higher number of genes than expected by chance. This phenomenon is not merely a consequence of tandem duplication. The genes with specific expression in testicular somatic cells are more abundant on the X chromosome, which favors the theory of accumulation of male-biased genes on the X chromosome. In contrast, the X-linked genes are under-represented in the transcriptome of total testis, which is in accordance with the idea of X-chromosome inactivation during the first meiotic division.

## Methods

### Tissue collection and RNA isolation

Mice were housed in specific pathogen free environment and their manipulation was in accordance with the Czech Animal Protection Act No. 246/92, 162/93, and decrees No. 311/97, fully compatible with the NIH Publication No. 85-23, revised 1985. Testes were obtained from 9-week-old males of the C57BL/6J mouse strain. The animals were killed by cervical dislocation; the testes were quickly removed from the body and released from tunica. The total RNA was extracted from homogenized testes using TRIzol (Invitrogen) according to the manufacturer's protocol. SAGE libraries were constructed from the total RNA isolated from both testes of a single male (TT 1) and from the pool consisting of equal weight amounts of total RNA isolated from both testes of three male littermates (TT 2).

### Construction of SAGE libraries, sequencing and tag extraction

SAGE libraries were constructed as described in the MicroSAGE protocol version 1.0e available from SAGE homepage [53] using *Nla*III as the anchoring enzyme and *Bsm*FI as the tagging enzyme. Two minor modifications of the MicroSAGE protocol were employed: the first strand cDNA synthesis reaction was incubated at 42°C and the amount of linkers used in the linker ligation step was decreased to ~10 ng. Sequencing was performed in a Beckmann Coulter CEQ 2000 DNA Analysis System. The sequence files were processed for the tag extraction using a custom Perl script. Tags were extracted only from clones containing > 2 ditags. Duplicated ditags, linker tags and all 1-bp linker variations were removed. Data of total testis SAGE libraries are available in the GEO repository [20] under accession numbers GSM34767 (TT 1) and GSM34768 (TT 2).

### Identification of SAGE tags

Tag identification to UniGene clusters was done using three methods: SAGEmap reliable mapping [22], Mouse SAGE Site reliable mapping [24] and RNA evidence mapping. The SAGEmap reliable mapping [23] uses a reliability score to classify tag-to-gene associations and tag-to-gene associations with the top two reliability scores are considered reliable. The Mouse SAGE Site [21] reliable mapping is based on the SAGEmap full mapping file and considers reliable the tag-to-gene associations that are supported by tags extracted from at least one mRNA sequence (from RefSeq, Mammalian Gene Collection, GenBank) or at least 3 ESTs with a poly(A) signal or at least 8 ESTs with no poly(A) signal. The RNA evidence mapping is also based on the SAGEmap full mapping file and considers reliable only tag-to-gene associations supported by tags extracted from at least one mRNA sequence. Mitochondrial tags were identified using all possible tags extracted from the mouse mitochondrial genome reference sequence [GenBank:NC_005089].

### Comparison of testis SAGE libraries

Tags significantly different between SAGE libraries were determined by Monte Carlo simulations. Using the described algorithm [54] a set of 100 000 random tables was generated keeping the row and column totals of the observed data fixed. For each tag, the proportion of simulations that produced a difference equal to or greater than the observed difference (p-chance) was computed. The set of 100 000 random tables was generated six times and the average p-chance was calculated. The fold factor was computed as the ratio of normalized tag counts in two SAGE libraries with values < 1 converted to reciprocal negatives. For the tags absent in one library a normalized tag count of single copy tags was assumed.

### Data sources

The SAGE library from somatic cells of the adult testis [19] was obtained from GEO repository [20], accession number GSM5435. Other SAGE libraries were obtained from GEO repository or downloaded from Internet sources (see Additional file 4 ). The data from the BDF1 testis SAGE library were obtained from a printed table in publication [32] (only the top 100 genes expressed in BDF1 testis are listed in publication, the whole library is currently not publicly available). Microarray data of mouse testis, generated by the GNF Mouse Atlas v2 project [33], were obtained from the hgFixed database of the UCSC Genome Browser [55,56].

### Hierarchical clustering of mouse SAGE libraries

Thirty-two mouse SAGE libraries constructed from bulk tissues (including normal and diseased) that were publicly available to date (July 1, 2004) were selected (see Additional file 4). For each pair of SAGE libraries a distance based on differences between normalized tag counts was computed [28]. The average agglomeration method was used in hierarchical clustering because of the highest cophenetic correlation (Pearson correlation between the observed distances and the distances calculated from the dendrogram).

### Selection and preparation of mouse SAGE libraries for genomic analysis

Twenty-seven SAGE libraries created from bulk tissues (excluding tumors) were organized into 7 groups by tissue type and tag counts from SAGE libraries within each group were combined (see Additional file 4). The groups of SAGE libraries include: brain (9 libraries, 329 745 tags), eye (6 libraries, 336 399 tags), heart (1 library, 84 275 tags), liver (2 libraries, 37 118 tags), kidney (6 libraries, 87 810 tags), limbs (2 libraries, 136 650 tags) and adipose tissue (1 library, 44 974 tags). These groups were analyzed in parallel with total testis (2 libraries, 76 854 tags) and adult testis somatic cells (1 library, 81 478 tags). All tags from prepared tissue groups, total testis and adult testis somatic cells SAGE libraries were identified to UniGene clusters using RNA evidence mapping (tag-to-gene association is supported by at least one mRNA sequence) and linked to LocusLink genes. Only tags with identification to a single LocusLink gene were subjected to further analysis. Tag counts from multiple tags matching the same LocusLink gene were combined.

### Distribution of tissue-specific genes on chromosomes

Analysis was done in parallel for testis-specific genes in total testis and somatic cells of adult testis. The tissue-specific genes were selected according to tag counts in the testis tissue and 7 non-testis tissues (see Additional file 4). A gene was considered to be tissue-specific if it was expressed only in one tissue and its expression was supported by tag count > 1. Each tissue-specific gene was then assigned to a chromosome (autosome or X chromosome) according to the LocusLink database and the group (testis or non-testis). The permutations algorithm performed 100 000 random shufflings of the chromosomes while keeping the sum of genes on autosomes and the X chromosome constant. The p-value (two tailed) was computed as doubled number of permutations yielding gene counts above/below (which of this was lower) or equal to the observed gene counts in testis tissue and the X chromosome.

### Identification of chromosomal clusters of genes with preferential expression in testis

The preferential expression measure (PEM) [31] was used to score differential expression of genes in testis tissues. PEM for total testis (PEM_TT_) and adult testis somatic cells (PEM_ATSC_) were calculated for each gene. The gene was considered to be preferentially expressed in total testis if PEM_TT_> = 1/2 PEM_TT(max)_, and in somatic cells of adult testis if PEM_ATSC_> = 1/2PEM_ATSC(max)_. PEM_(max) _values represent the maximum PEM value encountered in the tissue, PEM_TT(max) _= 1.169, PEM_ATSC(max) _= 1.145.

To prepare a tandem duplicate-free mouse genome we considered 19 684 known genes from the LocusLink database that were mapped on the mouse genome assembly (NCBI build 32) [57]. For each LocusLink gene, we obtained a known protein sequence (NP_ accessions) from the mouse RefSeq collection [58] and performed protein BLAST (standard settings) against the RefSeq known protein collection. The hits with expectation value < 1e^-10 ^and with an alignment of at least 50% length and 30% identity of the query sequence were processed and identified to LocusLink genes. If a LocusLink gene located in the vicinity of the original LocusLink gene was found among the hits (considering 10 adjacent genes in both directions), both genes were considered as a tandem duplicate pair and were excluded from the genome. As a result a tandem duplicate-free genome with 16 858 LocusLink genes was obtained.

Two sets of gene clusters with preferentially expressed genes were identified – for total testis and somatic cells of adult testis. All LocusLink genes from the tandem duplicate-free mouse genome were associated with the expression status (preferentially expressed, expressed, unknown). Each chromosome was searched using a sliding window of three adjacent genes and three consecutive preferentially expressed genes were considered as a cluster (tight clusters). Another search was performed using a sliding window of six adjacent genes and at least three preferentially expressed genes were required to form a cluster spanning from the first to the last preferentially expressed gene (loose clusters). The overlapping clusters were merged into a single cluster encompassing all involved genes (separately for tight or loose clusters). The permutations performed 100 000 random shufflings of the expression status in the genome while keeping the gene positions constant. A search with the above defined sliding windows determined the number of preferentially expressed genes located in clusters in each randomized genome. The p-value (one tailed) was computed as the number of permutations yielding greater than or equal to the observed number of preferentially expressed genes located in clusters.

### Statistical evaluation

All statistical analyses, including Monte Carlo simulations, hierarchical clustering, chromosomal and gene permutations were conducted in R statistical environment [59] using custom scripts.

### Database versions

The following database versions were used in all analyses: Mouse UniGene build #136 (March 26, 2004), mouse SAGEmap (April 3, 2004) corresponding to the mouse UniGene #136, LocusLink (April 3, 2004), mouse genome assembly NCBI build 32 (November 2003), mouse Reference Sequence collection (April 3, 2004) and Gene Ontology database (July, 2004).

## Authors' contributions

PD constructed the SAGE libraries and performed the bioinformatic analyses. CV carried out sequencing of SAGE libraries. PS participated in bioinformatic analysis of gene order. VP is Head of the Center for Integrated Genomics. JF conceived the study and coordinated work. PD and JF wrote the article. All authors read and approved the final manuscript.

## Supplementary Material

Additional File 1 **SAGE tags detected in mouse total testis**. List of 7 481 tags with tag count > 1 in combined total testis SAGE library with reliable tag identification according to the Mouse SAGE Site database. Click here for file

Additional File 2 **Tags with significantly different tag counts between total testis and adult testis somatic cells**. List of 3 258 tags with significantly different tag counts between the total testis and adult testis somatic cells SAGE libraries determined by Monte Carlo simulations (1 691 tags have increased tag counts in total testis, 1 567 tags have increased tag counts in adult testis somatic cells at p-chance < 0.05). The reliable tag identification according to the Mouse SAGE Site database is provided. Click here for file

Additional File 3 **Genes with predominant expression in germinal and somatic cells of the testis**. List of 924 and 802 genes with predominant expression in germinal and somatic cells of the testis, respectively, based on the comparison of total testis and adult testis somatic cells SAGE libraries. Tags with significantly different tag counts (p-chance < 0.05, Monte Carlo simulation) and at least five-fold increased/decreased tag counts were selected and identified using RNA evidence mapping. Click here for file

Additional File 4 **Mouse SAGE libraries used in genomic analysis and hierarchical clustering**. List of mouse SAGE libraries publicly available to date July 1, 2004 that were used in genomic analysis and hierarchical clustering. Click here for file

Additional File 5 **Genes specific for total testis or adult testis somatic cells**. List of 395 and 81 genes with specific expression in total testis or adult testis somatic cells determined by comparison to SAGE data from seven non-testis tissues (brain, eye, heart, liver, kidney, limbs and adipose tissue). A gene was considered to be testis specific if the corresponding tags were present only in total testis or adult testis somatic cells SAGE libraries and missing in all non-testis libraries. Tags were identified using RNA evidence mapping. Click here for file

Additional File 6 **Testis expressed genes located on the X chromosome (summary)**. Summary of the X-linked genes expressed in total testis and adult testis somatic cells. Tags were identified using RNA evidence mapping. Click here for file

Additional File 7 **Preferentially expressed genes in total testis and testicular somatic cells**. Preferentially expressed genes were determined separately for total testis and adult testis somatic cells in conjunction with their expression in seven non-testis tissues. Expression of genes was scored using preferential expression measure (PEM). A gene was considered to be preferentially expressed if PEM score was above 50 % of the maximum PEM value encountered in that tissue. Tags were identified using RNA evidence mapping. Click here for file

Additional File 8 **Genes preferentially expressed in testis located in chromosomal clusters within tandem duplicate-free genome**. Chromosomal clusters of genes preferentially expressed in testis were localized by the search with a sliding window. Two types of clusters were identified: tight clusters (containing at least three adjacent preferentially expressed genes in testis) and loose clusters (containing at least three preferentially expressed genes in testis among the six adjacent genes). Click here for file

Additional File 9 **Manual comparison of the most highly expressed genes in three total testis transcriptomes (GNF atlas, B6 testis, BDF1 testis)**. The list of 35 most highly expressed genes in total testis according to the GNF Mouse Atlas v2 organized with the appropriate *Nla*III and *Rsa*I SAGE tags extracted from their representative mRNA/RefSeq sequences. Click here for file
